# Emotional Response to Various Exercise Types in Patients With Mental Disorders

**DOI:** 10.7759/cureus.75371

**Published:** 2024-12-09

**Authors:** Miyuki Nemoto, Kiyotaka Nemoto, Hiroyuki Sasai, Miho Ota, Maiko Haneda, Aya Sekine, Tetsuaki Arai

**Affiliations:** 1 Department of Psychiatry, University of Tsukuba, Tsukuba, JPN; 2 Research Team for Promoting Independence and Mental Health, Tokyo Metropolitan Institute for Geriatrics and Gerontology, Itabashi, JPN; 3 Department of Neuropsychiatry, University of Tsukuba, Tsukuba, JPN; 4 Department of Psychiatry, University of Tsukuba Hospital, Tsukuba, JPN

**Keywords:** emotion, emotional response, exercise types, mental disorders, yoga

## Abstract

Objectives

This study examined the relationship between different types of exercise bouts and emotional responses in patients with mental disorders.

Methods

This study utilized an acute pre-/post-interventional design. Patients participated in six types of exercises: yoga, strength training, dual-task exercises, aerobic exercises, multicomponent exercises, and dance. These sessions were conducted for 60 minutes per day, once a week, from June 2018 to February 2019. Emotional states, including pleasantness, relaxation, and anxiety, were evaluated using the Mood Check List-Short Form 2 before and after each session.

Results

Twenty-four patients with mental disorders, including mood disorders, schizophrenia, and other conditions, aged 20-77 years, participated in a total of 272 sessions across six exercise types. Significant emotional changes were observed before and after the exercises, with an increase in pleasant feelings and a decrease in unpleasant feelings for all exercise types except the dual-task exercises. Yoga, in particular, showed large effect sizes for emotional changes, ranging from 0.65 to 0.72.

Conclusions

Yoga was found to enhance pleasantness and relaxation while reducing anxiety, whereas dual-task exercises appeared less effective in providing these benefits. These findings can help inform the selection of effective exercise methods for patients with mental disorders.

## Introduction

A growing body of evidence highlights the benefits of exercise in the treatment of mental disorders. Clinical practice guidelines in the United States, United Kingdom, and Australia recommend exercise as part of depression treatment [1-3]. Research reviews consistently demonstrate the generally positive effects of exercise on mental health [4]. Among the various forms of exercise, aerobic activities such as jogging, swimming, and cycling have been extensively studied and shown to improve depression and anxiety, with therapeutic effects comparable to those of conventional pharmacotherapy [5]. Additionally, yoga, which integrates physical postures with mindfulness and relaxation, has been found to alleviate symptoms of anxiety and depression [6]. Sports-based approaches, including team sports and recreational activities, have also been reported to enhance mental health [7]. Vocational arts and dance-based exercises, such as creative dance and structured movement therapies, have demonstrated promise in fostering emotional expression, reducing stress, and improving quality of life [8].

While these studies report positive emotional responses to individual exercises, reviews comparing the effects of different types of exercise on mental disorders have consistently highlighted the mental health benefits of physical activity. For example, aerobic exercises have been shown to have particularly significant positive effects on mood and overall mental well-being.

However, no study has directly compared multiple exercise types within the same cohort of patients to determine how individuals with mental disorders emotionally respond to each type of exercise (positively or negatively). This leaves a gap in understanding the specific emotional responses elicited by different exercise modalities when assessed within the same group of patients.

To address this, the present study investigated the relationship between exercise type and emotional responses in patients with mental disorders. By examining multiple exercise types within the same cohort, we aimed to assess the diverse emotional responses each exercise type evoked. Understanding the relationship between exercise modalities and emotional outcomes - such as identifying exercises that alleviate mood disturbances or intensify anxiety - is crucial for selecting training methods that maximize the therapeutic effects of exercise on patients with mental disorders.

This study was conducted as part of the exercise program of the Return-to-Work Day Care (RWDC) for patients with mental disorders at the University of Tsukuba Hospital in Tsukuba, Ibaraki, Japan.

## Materials and methods

Study design and setting 

**Figure 1:**
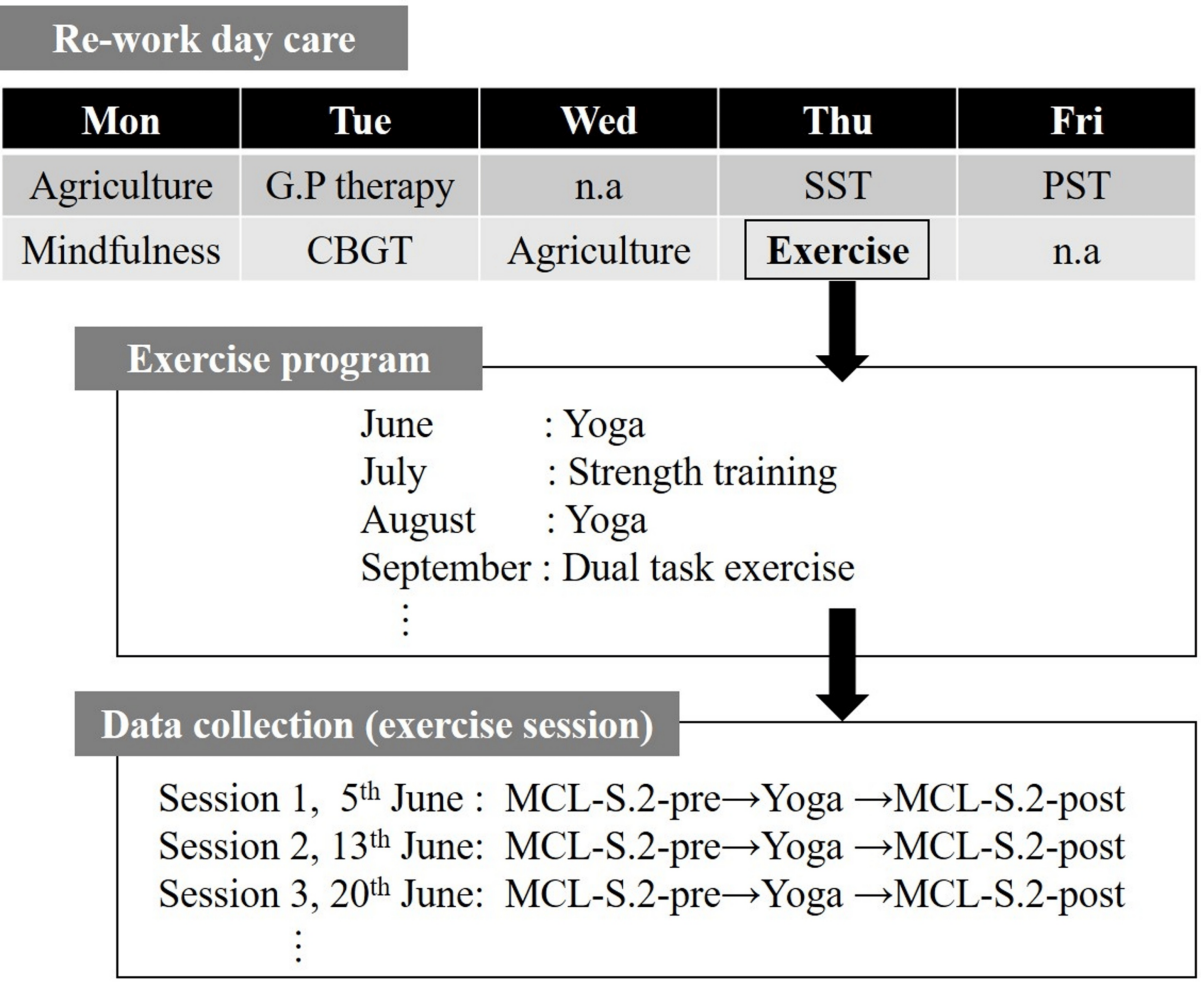
Study setting This study utilized the exercise program, a component of the continuous RWDC initiative aimed at supporting the return to work for patients with mental disorders. The study specifically focused on the exercise program within the RWDC and employed an acute pre- and post-intervention design. Data were collected before and after each exercise session conducted as part of the RWDC. CBGT: cognitive behavior therapy; G.P therapy: group psychotherapy; n.a: not available; PST: problem-solving therapy; SST: social skills training; MCL-S.2: Mood Check List-Short Form 2; RWDC: Return-to-Work Day Care

This study was conducted from June 2018 to February 2019 as part of the exercise program within the continuous RWDC initiative at the University of Tsukuba Hospital, located in Tsukuba City, Ibaraki, Japan (Figure 1). The RWDC program was designed to support patients with mental disorders in returning to work and was held three to five days per week at the hospital’s fitness gym. The sessions were led by specialized instructors, occupational therapists, and nurses.

The RWDC offers a variety of activities, including agriculture, cognitive-behavioral therapy, and group psychotherapy. This study specifically focused on the exercise component of the RWDC program, employing an acute pre- and post-intervention design. Data were collected immediately before and after each exercise session as part of the routine activities within the RWDC.

The study protocol adhered to the principles outlined in the Declaration of Helsinki and received approval from the Ethics Committee of the University of Tsukuba Hospital (approval number R06-025). Informed consent was obtained from all participating patients.

Participants

Patients were included if they (1) met the Diagnostic and Statistical Manual of Mental Disorders (DSM-5) criteria [2], (2) were diagnosed with mental disorders such as mood disorders (bipolar disorder and major depressive disorder), schizophrenia, adjustment disorder, autism spectrum disorder, or somatic symptom disorder (with mild to moderate symptom severity) by trained attending psychiatrists (TA, MO, and KN) during their application to the RWDC program, with diagnosis determined through a clinical interview, and (3) participated in the RWDC program between April 2013 and November 2018. Patients were excluded if they had (1) a terminal illness or (2) major musculoskeletal or cardiovascular conditions, or lacked medical clearance.

The exercise program

Patients participated in six types of exercise: yoga, strength training, dual-task exercises, aerobic exercise, multicomponent exercises (including recreation), and dance. The sessions included yoga (three modules, 12 sessions), strength training (one module, five sessions), dual-task exercises (one module, four sessions), aerobic exercise (one module, four sessions), multicomponent exercises (two modules, nine sessions), and dance (one module, three sessions). All sessions were led by an exercise instructor at a medically supervised university hospital, held weekly for 60 minutes per day, from June 2018 to February 2019. Each session consisted of a five-minute warm-up, a 50-minute main exercise, and five minutes of relaxation. Table 1 provides additional details of the exercise program. To promote engagement and gradual physical adaptation, the order of exercises was designed to alternate between low-intensity sessions (e.g., yoga) and high-intensity sessions (e.g., strength training or aerobic exercise). This approach aligns with previous research suggesting that combining varying intensities in physical exercise optimizes mental health outcomes by enhancing overall mood stability [3]. All exercises were performed at moderate intensity, corresponding to a rate of perceived exertion (RPE) of 11-13 [9].

**Table 1:** Exercise program

Menu	Contents
Yoga	Three yoga lessons (one type of lesson/module). Examples: relax yoga, power yoga, hatha yoga (primarily focused on hatha yoga, with elements from power yoga and relaxation-focused yin yoga incorporated). The core asanas practiced during all sessions included Tadasana (mountain pose), Vrikshasana (tree pose), Trikonasana (triangle pose), Bhujangasana (cobra pose), Paschimottanasana (seated forward bend), and Shavasana (corpse pose). Additionally, session-specific asanas were included. For example, hatha yoga sessions incorporated Virabhadrasana I (warrior pose I) and Virabhadrasana II (warrior pose II). Pranayama and Dhyana: focused on basic abdominal breathing and relaxation.
Strength training	Five strength lessons (one type of lesson/session). Examples: upper limbs: arm curls, shoulder press (using a machine), trunk: plank, hip lift, and lower limbs: squats, leg curls (using a machine). Each session included exercises targeting the upper limbs, trunk, and lower limbs to ensure a balanced workout. Participants performed 10 repetitions per set, repeated for a total of five sets for each muscle group.
Dual-task exercise	Four dual-task exercise lessons (one type of lesson/session). Examples: calculate while stepping in place, clap on multiples of three while stepping in place, use a ladder, and walk according to certain rules.
Aerobic exercise	Four aerobic exercise lessons (one type of lesson/session). Examples: low impact, high impact, using step platform, and steps (march, jog, skip, knee lift, kick, jack, and lunge, etc.)
Multicomponent exercise	Two multicomponent exercise lessons (one type of lesson/module). Examples: circuit workout, recreation.
Dance	Three dance lessons (one type of lesson/session). Examples: folk dance (music: Genghis Khan, Oklahoma Mixer, and Macarena), rhythm dance (music: Japanese popular music), and creative dance (participants were divided into small groups, and each group selected its own music).

Outcome assessments

Measurements

Trained staff, including an occupational therapist and nurse, measured patients' height and weight in light clothing without shoes in May 2018, before the start of the exercise program. BMI (kg/m²) was then calculated. Psychiatrists assessed the duration of the mental disorders, work absence, and medical history.

Emotional assessments

The Mood Check List-Short Form 2 (MCL-S.2) is used to assess emotional changes associated with exercise [10]. It includes three components: pleasantness, relaxation, and anxiety, with each component comprising four items. Patients responded using a seven-point scale (1 = strongly disagree; 7 = strongly agree). Scores for each component range from 4 to 28, with higher scores for pleasantness and relaxation, and lower scores for anxiety, indicating a more favorable emotional state. The MCL-S.2 has demonstrated good reliability (Cronbach α = 0.84 or higher) and construct validity. It was administered before and after each exercise session within 10 minutes.

Statistical analysis

Basic characteristics, such as sex and diagnosis, were reported as frequencies, while other characteristics were presented as means and SDs. Wilcoxon signed-rank tests were used to examine changes in emotions before and after each exercise session. Pre-post changes in the MCL-S.2 were expressed as medians, p-values, and effect sizes (r). Effect sizes were categorized as small (0.10-0.29), medium (0.30-0.49), and large (≥0.5) [11]. Stratified analysis was conducted to examine emotional changes based on different diagnoses, including mood disorder and schizophrenia. Data were analyzed using IBM SPSS Statistics for Windows, Version 28.0 (Released 2021; IBM Corp., Armonk, NY, USA).

## Results

**Figure 2:**
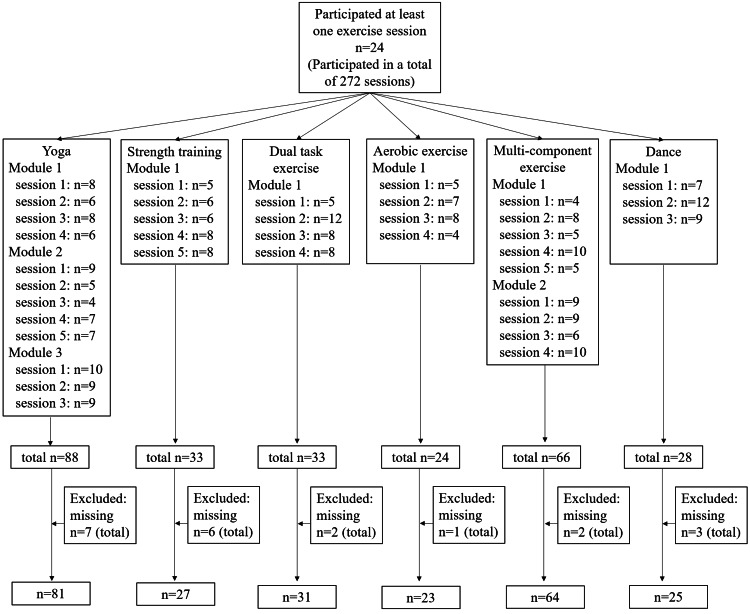
Flowchart of study patients

Figure 2 illustrates the study’s flowchart. A total of 24 patients participated in at least one exercise session, completing 272 sessions. Only patients with complete data for each session were included in the final analysis. The number of patients analyzed per exercise modality were as follows: yoga (n = 81), strength training (n = 27), dual-task exercise (n = 31), aerobic exercise (n = 23), multicomponent exercise (n = 64), and dance (n = 25).

Table 2 provides the patient characteristics. The study included 24 adults with mental disorders (seven women), aged 20-77 years, with a mean age of 39.5 (±12.2) years. Diagnoses included mood disorders (12 patients), schizophrenia (five patients), and other disorders (seven patients).

**Table 2:** Characteristics of the patients Data are shown as mean (SD) for continuous variables. ^a^ Missing: two patients ^b^ Missing: one patient ^c^ Never worked: eight patients ^d^ Previously diagnosed conditions and conditions identified within physician screen at baseline assessment ^e^ Number of medications including prescribed supplements ^f^ Missing: four patients

Demographics and anthropometrics	N = 24
Sex	
Male	17
Female	7
Age, years	39.5 (12.2)
Height, cm	167.5 (5.8)^a^
Weight, kg	67.3 (13.9)^a^
BMI kg/m^2^	24.0 (4.6)^a^
Diagnosis, n	
Mood disorder	12
Schizophrenia	5
Other	7
Duration, year	10.5 (7.5)^b^
Period of absence from work, year	3.1 (4.7)^c^
Medical history	
Diagnosed comorbidities, n^d^	0.3 (0.5)
Prescribed medications, n^e^	5.1 (3.4)^f^

**Figure 3:**
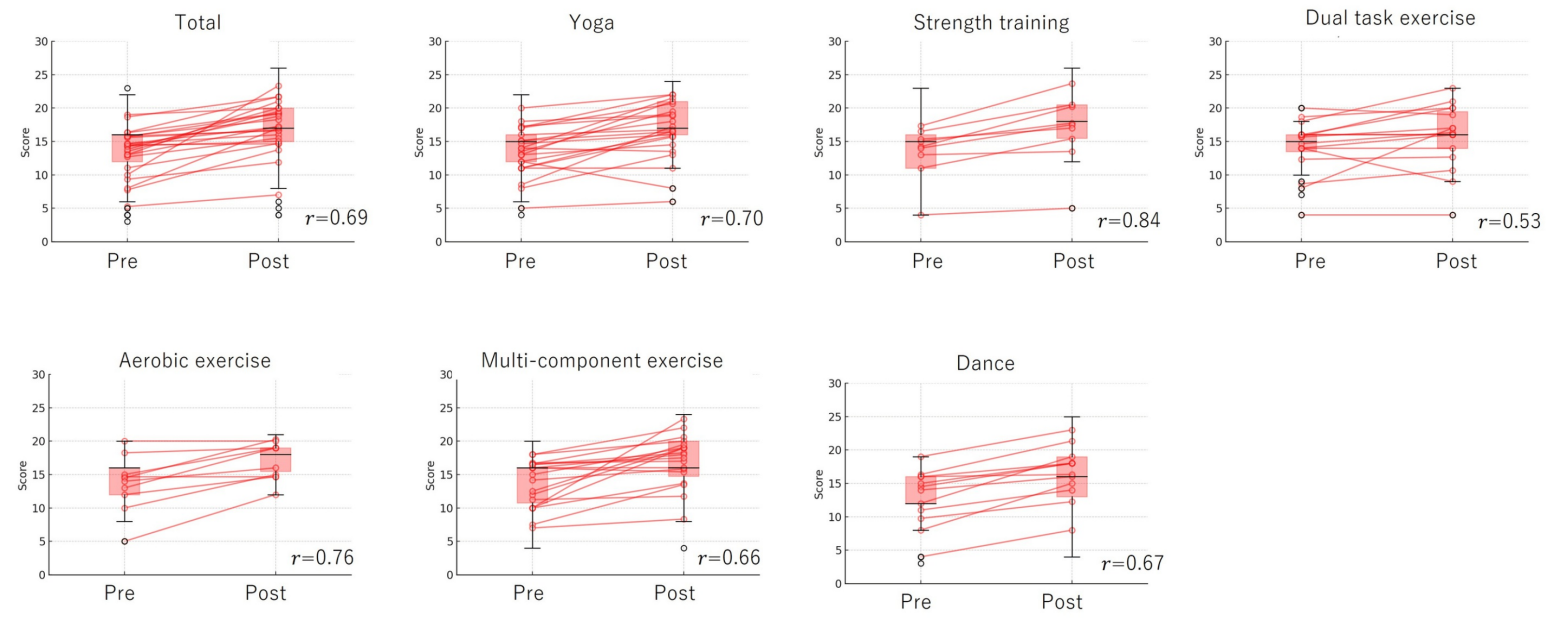
Changes in MCL-S.2 before and after exercise sessions by type (pleasantness) This figure illustrates the changes in psychological scores — specifically, pleasantness, relaxation, and anxiety — before and after various exercise interventions. Each line represents the individual participant data, showing score changes from the pre-exercise to post-exercise phase. Overlaid box plots show the overall score distribution, highlighting central tendencies and variability within each exercise type and psychological measure. The results are presented in red. The effect size (r) is indicated, with effect sizes classified as small (0.10-0.29), medium (0.30-0.49), and large (≥0.5). MCL-S.2: Mood Check List-Short Form 2

**Figure 4:**
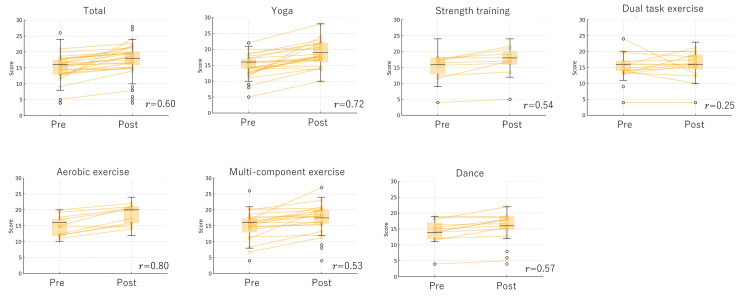
Changes in MCL-S.2 before and after exercise sessions by type (relaxation) This figure illustrates the changes in psychological scores — specifically, pleasantness, relaxation, and anxiety — before and after various exercise interventions. Each line represents individual participant data, showing score progression from the pre-exercise to the post-exercise phase. Overlaid box plots display the overall score distribution, indicating central tendencies and variability within each exercise type and psychological measure. The results are represented in yellow. The effect size (r) is provided, with effect sizes categorized as small (0.10-0.29), medium (0.30-0.49), and large (≥0.5). MCL-S.2: Mood Check List-Short Form 2

**Figure 5:**
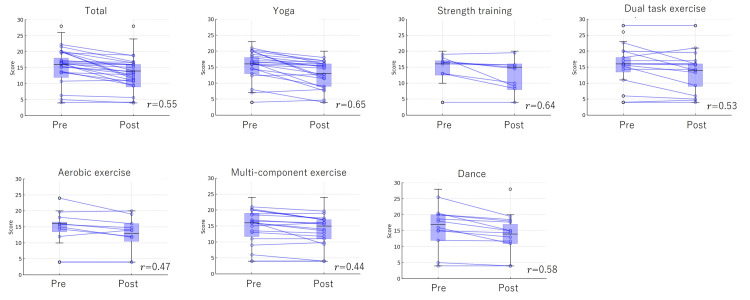
Changes in MCL-S.2 before and after exercise sessions by type (anxiety) This figure illustrates the changes in psychological scores — specifically, pleasantness, relaxation, and anxiety — before and after various exercise interventions. Each line represents individual participant data, showing the progression of scores from the pre-exercise to the post-exercise phase. Box plots overlay each group, displaying the overall score distribution and indicating central tendencies and variability within each exercise type and psychological measure. The results are represented in blue. The effect size (r) is provided, with effect sizes categorized as small (0.10-0.29), medium (0.30-0.49), and large (≥0.5). MCL-S.2: Mood Check List-Short Form 2

Figure 3, Figure 4, and Figure 5 illustrate changes in MCL-S.2 scores before and after exercise sessions, categorized by exercise type. Changes in psychological dimensions - pleasantness, relaxation, and anxiety - were measured before and after each exercise intervention. The individual trajectories of each participant were tracked from pre- to post-exercise, highlighting variations in psychological responses across different exercise types. Overlaid box plots depict the distribution of scores, emphasizing trends in central tendency and variability for each exercise type and emotional dimension. This approach offers insights into both individual and group-level responses to the exercise interventions. Significant emotional changes were observed across all exercises, with pleasant feelings increasing and unpleasant feelings decreasing post-exercise. Yoga, in particular, demonstrated a large effect size for all emotional changes (0.65-0.72). For strength training, pleasantness, relaxation, and anxiety showed large effect sizes (0.84, 0.54, 0.63, respectively). Dual-task exercise showed large effect sizes for pleasantness and anxiety (0.52, 0.53), with a small effect size for relaxation (0.25). Aerobic exercise resulted in large effect sizes for pleasantness and relaxation (0.76, 0.80), while anxiety showed a medium effect size (0.47). Multicomponent exercise demonstrated large effect sizes for pleasantness and relaxation (0.66, 0.53), and a medium effect size for anxiety (0.44). Dance also showed large effect sizes for pleasantness, relaxation, and anxiety (0.67, 0.57, 0.58, respectively).

We evaluated the effects of various exercise types on emotional changes in patients with mood disorders, schizophrenia, and other mental illnesses (Table 3, Table 4, Table 5). In the mood disorder group (Table 3), significant improvements in pleasantness and relaxation were observed across different exercise types, with overall pleasantness showing a large effect (r = 0.67). Aerobic exercise (r = 0.82) and dance (r = 0.74) particularly demonstrated strong effects on enhancing pleasantness. For anxiety, yoga and dance were notably effective in reducing symptoms (r = 0.70 and r = 0.75, respectively). In the schizophrenia group (Table 4), significant improvements were seen across all emotional domains-pleasantness, relaxation, and anxiety. Aerobic exercise (r = 0.90) and multicomponent exercise (r = 0.92) showed strong effects in improving pleasantness and relaxation. In the group with other mental illnesses (Table 5), improvements in pleasantness and relaxation were observed across various exercise types. Specifically, aerobic exercise (pleasantness: r = 0.88, relaxation: r = 0.88) and yoga (pleasantness: r = 0.82, relaxation: r = 0.84) demonstrated positive emotional effects. Additionally, multicomponent exercise was the most effective in reducing anxiety (r = 0.84).

**Table 3:** Emotional changes by exercise types in mood disorder Wilcoxon signed-rank sum tests were used to calculate p-values. MCL-S.2: Mood Check List-Short Form 2

Outcomes	Total, n	Median (Pre)	Median (Post)	p-value	Effect size (r)
MCL-S.2; Pleasantness					
Total	101	15.0	17.0	0.00	0.67
Yoga	32	15.0	17.0	0.00	0.63
Strength training	7	14.0	18.0	0.05	0.75
Dual-task exercise	12	15.0	15.5	0.06	0.55
Aerobic exercise	5	16.0	18.0	0.07	0.82
Multicomponent exercise	32	15.5	17.0	0.00	0.68
Dance	13	12.0	16.0	0.01	0.74
Relaxation					
Total	101	16.0	18.0	0.00	0.57
Yoga	32	16.0	18.0	0.00	0.61
Strength training	7	14.0	20.0	0.50	0.26
Dual-task exercise	12	15.0	16.0	0.67	0.13
Aerobic exercise	5	17.0	20.0	0.04	0.93
Multicomponent exercise	32	16.0	18.0	0.00	0.57
Dance	13	14.0	16.0	0.01	0.75
Anxiety					
Total	101	16.0	15.0	0.00	0.46
Yoga	32	17.0	13.5	0.00	0.70
Strength training	7	17.0	11.0	0.02	0.90
Dual-task exercise	12	16.5	15.5	0.03	0.64
Aerobic exercise	5	14.0	11.0	0.20	0.58
Multicomponent exercise	32	17.0	15.0	0.02	0.40
Dance	13	19.0	16.0	0.01	0.75

**Table 4:** Emotional changes by exercise types in schizophrenia Wilcoxon signed-rank sum tests were used to calculate p-values. MCL-S.2: Mood Check List-Short Form 2

Outcomes	Total, n	Median (Pre)	Median (Post)	p-value	Effect size (r)
MCL-S.2; Pleasantness					
Total	31	16.0	20.0	0.00	0.77
Yoga	12	16.0	21.0	0.05	0.82
Strength training	3	16.0	24.0	0.11	0.92
Dual-task exercise	7	16.0	20.0	0.11	0.64
Aerobic exercise	6	15.0	19.5	0.03	0.90
Multicomponent exercise	3	16.0	19.0	0.18	0.92
Dance	1	na	na	na	na
Relaxation					
Total	31	16.0	21.0	0.00	0.79
Yoga	12	16.0	22.0	0.05	0.81
Strength training	3	16.0	22.0	0.10	0.92
Dual-task exercise	7	16.0	20.0	0.07	0.76
Aerobic exercise	6	16.0	20.5	0.03	0.90
Multicomponent exercise	3	16.0	20.0	0.17	0.93
Dance	1	na	na	na	na
Anxiety					
Total	31	16.0	11.0	0.00	0.79
Yoga	12	14.0	9.5	0.05	0.81
Strength training	3	13.0	8.0	0.11	0.93
Dual-task exercise	7	16.0	9.0	0.03	0.83
Aerobic exercise	6	16.0	12.5	0.03	0.90
Multicomponent exercise	3	16.0	16.0	0.17	0.93
Dance	1	na	na	na	na

**Table 5:** Emotional changes by exercise types in other mental illness Wilcoxon signed-rank sum tests were used to calculate p-values. MCL-S.2: Mood Check List-Short Form 2

Outcomes	Total, n	Median (Pre)	Median (Post)	p-value	Effect size (r)
MCL-S.2; Pleasantness					
Total	119	15.0	16.0	0.00	0.72
Yoga	37	13.0	16.0	0.00	0.82
Strength training	17	15.0	17.0	0.00	0.88
Dual-task exercise	12	15.5	16.0	0.12	0.73
Aerobic exercise	12	16.0	16.0	0.01	0.88
Multicomponent exercise	29	16.0	16.0	0.00	0.83
Dance	12	14.0	16.5	0.03	0.73
Relaxation					
Total	119	16.0	16.0	0.00	0.74
Yoga	37	16.0	18.0	0.00	0.84
Strength training	17	16.0	17.0	0.01	0.73
Dual-task exercise	12	15.5	16.0	0.88	0.50
Aerobic exercise	12	16.0	16.0	0.01	0.88
Multicomponent exercise	29	16.0	16.0	0.01	0.67
Dance	12	14.5	16.0	0.20	0.54
Anxiety					
Total	119	16.0	15.0	0.00	0.78
Yoga	37	16.0	16.0	0.00	0.78
Strength training	17	16.0	15.0	0.16	0.80
Dual-task exercise	12	15.5	15.5	0.91	0.66
Aerobic exercise	12	15.0	15.0	0.59	0.69
Multicomponent exercise	29	16.0	14.0	0.01	0.84
Dance	12	12.0	12.0	0.20	0.78

## Discussion

This study examined the relationship between exercise type and emotions in patients with mental disorders. Exercise was found to improve pleasantness and relaxation, with yoga particularly enhancing relaxation and reducing anxiety. However, dual-task exercise did not significantly improve pleasantness or relaxation. Other forms of exercise did not produce significant reductions in anxiety. Overall, while yoga had a positive impact on emotions, dual-task exercise and strength training demonstrated more modest effects.

Mental disorders and exercise and their types

This study indicates that exercise for patients with mental disorders may enhance feelings of pleasantness and relaxation and reduce anxiety. Exercise interventions for individuals with mental disorders have been shown to improve mood and emotional well-being [4]. Previous studies have demonstrated that certain types of exercise, such as yoga, aerobic exercise, and strength training, are particularly effective in enhancing mood and reducing symptoms of anxiety and depression [4,5]. Consistent with these findings, our study revealed substantial effect sizes for these exercise types (0.69-0.83), indicating their therapeutic benefits for emotional health.

Consistent with previous studies, the results of this study demonstrated that exercise positively impacted the emotions of patients with mental disorders, with yoga producing notable emotional changes. The alignment of yoga’s effects with prior findings can be attributed to its multifaceted nature, encompassing physical, mental, and emotional components. Yoga is a comprehensive practice that combines physical postures, breathing techniques, relaxation, and meditation [12]. Both meditation and exercise have been shown to effectively reduce symptoms of depression [12,13], and the integration of multiple elements in yoga may offer advantages over isolated practices, such as meditation or exercise alone. While this study did not incorporate intensive or advanced meditation/dhyana practices, elements of these techniques were integrated into the sessions. It is likely that the inclusion of meditation/dhyana contributed to the reduction in anxiety levels among participants. Moreover, yoga can improve exercise adherence and may serve as an alternative for individuals hesitant to participate in traditional exercise forms [14]. Previous reviews and meta-analyses have also highlighted yoga's positive effects on relieving depressive symptoms in people with mental disorders [12]. The positive aspects of yoga, such as its multifaceted components, its role as an alternative form of exercise, and its impact on mental health, may have contributed to its beneficial effects on emotions.

Aerobic exercise has consistently been shown to improve mood and alleviate symptoms of depression and anxiety, likely due to its ability to increase endorphins and other neurochemicals involved in mood regulation [15]. Additionally, the rhythmic and repetitive movements in aerobic activities promote relaxation and induce a “flow” state, further enhancing mental well-being [16].

Although traditionally associated with physical health benefits, strength training has also been linked to improved mood and reduced anxiety. A meta-analysis by Gordon et al. [17] found that strength training positively influenced psychological health, possibly through mechanisms related to increased self-efficacy and improved body image, which contribute to relaxation and stress reduction.

The present study found that yoga, strength training, and aerobic exercise had more pronounced positive effects on pleasantness and relaxation compared to other exercise types. These findings align with existing literature, supporting the inclusion of these exercise types in programs aimed at optimizing emotional well-being for individuals with mental health conditions.

In contrast, dual-task exercises, which involve both cognitive and motor tasks, may create challenges. In a dual-task scenario, if a motor task and a cognitive task compete for limited cognitive resources, the performance of one or both tasks may be disrupted (dual-task interference) [18]. Cognitive impairment is a key feature in patients with mental disorders, and its prevalence presents a significant challenge [19]. Cognitive impairment affects approximately 45% of individuals with schizophrenia, 64% with bipolar disorder, and 77% with major depressive disorder [20]. Thus, it can be inferred that dual-task interference may be more pronounced in patients with mental disorders compared to healthy individuals, potentially preventing them from experiencing pleasantness or relaxation due to the high cognitive load. This study highlighted the possibility that dual-task exercises might induce negative emotions in patients with mental disorders, providing new insights into the role of cognitive limitations on the emotional impact of exercise in this population.

Numerous studies have demonstrated that exercise can reduce anxiety [21]. In this study, significant reductions in anxiety were observed across all exercise categories, although the effects were generally small. High-intensity exercise is known to cause temporary discomfort [4], and while the exercise was performed at an RPE of 11-13, the actual intensity may have varied.

Clinical and research implications

From a clinical perspective, it is essential to design exercise programs for patients with mental disorders that consider emotional responses. Suwabe et al. [22] demonstrated that positive moods during exercise can improve executive function, suggesting that activities that promote positive emotions and reduce anxiety may be especially beneficial for exercise programs targeting individuals with mental disorders.

This study highlights that emotional effects vary by exercise type. Given the distinct pathologies and etiologies of mental disorders [23,24], these conditions respond differently to the same exercise interventions. For example, yoga was shown to have a particularly strong positive impact on relaxation and anxiety reduction in this study. Yoga’s multifaceted approach, which combines physical postures, controlled breathing techniques, and structured relaxation, makes it especially effective in addressing various emotional and psychological needs. The integration of these elements promotes not only physiological benefits - such as reduced stress levels and enhanced parasympathetic activity - but also mental calm and emotional stability. These characteristics make yoga highly adaptable for treating various mental health conditions, including anxiety disorders and depression.

Moreover, previous research has highlighted the most effective exercise types for specific psychiatric disorders. For example, multimodal exercise has been identified as particularly beneficial for individuals with depression [25]. In this study, multimodal exercise also demonstrated a moderately positive effect on emotional outcomes. Future research should focus on identifying the most effective combinations of exercise types tailored to different mental health conditions to optimize emotional and psychological benefits.

Strengths and limitations

This study’s strength lies in its exploration of emotional responses to various exercise types in patients with mental disorders. However, it has several limitations.

First, the small sample size and absence of a control group limited our ability to assess the true effectiveness of the intervention. Future studies should increase the sample size to address this limitation, though determining these effects with certainty remains challenging. Additionally, evaluating how changes over time and factors such as learning effects may influence outcomes presents another challenge.

Second, the lack of examiner or evaluator blinding could have introduced bias in evaluations and assessments. This issue should be addressed in future studies to improve the reliability of the findings.

Third, the study used the RPE to measure exercise intensity; however, the data on patients’ individual RPE scores were unavailable, potentially leading to variations in exercise intensity settings.

Fourth, while patient preferences may influence the perceived benefits of exercise, we did not assess individual preferences for exercise or music. Although a subjective questionnaire was used, it did not specifically capture these preferences, which may have impacted their emotional responses. Future studies should implement strategies to directly capture patient preferences, such as pre-intervention surveys or post-session feedback forms.

Fifth, the study lacked detailed data on medication changes during the study period. Although all patients were under the care of psychiatrists, some were treated outside our institution, which limited our ability to track medication adjustments comprehensively. Since changes in psychiatric medications can significantly affect emotional and psychological outcomes, future research should systematically track medication changes to evaluate their influence on exercise-related effects.

Lastly, the possibility of carry-over effects exists in this study. The exercises were part of a larger RWDC program, so a preceding program could have influenced the results. Additionally, emotions related to the previous week’s exercise may have carried over. Therefore, future research should consider conducting a separate transient exercise intervention to examine the effects independently of the RWDC program.

## Conclusions

Yoga, in particular, has been shown to enhance pleasantness and relaxation while reducing anxiety. In contrast, dual-task exercises may be less effective in promoting pleasantness and relaxation. Furthermore, other exercise modalities may not significantly reduce anxiety. These findings are crucial for developing tailored exercise programs aimed at improving emotional well-being in individuals with mental disorders.
